# Review of Natural Product-Derived Compounds as Potent Antiglioblastoma Drugs

**DOI:** 10.1155/2017/8139848

**Published:** 2017-10-18

**Authors:** Moon Nyeo Park, Hyo Sook Song, Myungsun Kim, Min-Jung Lee, Whisung Cho, Hyun-Jin Lee, Cho-Hyun Hwang, Soojong Kim, Yechae Hwang, Beomku Kang, Bonglee Kim

**Affiliations:** ^1^College of Korean Medicine, Kyung Hee University, Seoul, Republic of Korea; ^2^Department of Science in Korean Medicine, College of Korean Medicine, Graduate School, Kyung Hee University, Seoul, Republic of Korea

## Abstract

Common care for glioblastoma multiforme (GBM) is a surgical resection followed by radiotherapy and temozolomide- (TMZ-) based chemotherapy. Unfortunately, these therapies remain inadequate involving severe mortality and recurrence. Recently, new approaches discovering combinations of multiple inhibitors have been proposed along with the identification of key driver mutations that are specific to each patient. To date, this approach is still limited by the lack of effective therapy. Hopefully, novel compounds derived from natural products are suggested as potential solutions. Inhibitory effects of natural products on angiogenesis and metastasis and cancer suppressive effect of altering miRNA expression are provident discoveries.* Angelica sinensis* accelerates apoptosis by their key substances influencing factors of apoptosis pathways. Brazilin displays antitumor features by making influence on reactive oxygen species (ROS) intensity.* Sargassum serratifolium*, flavonoids, and so on have antimetastasis effect.* Ficus carica* controls miRNA that inhibits translation of certain secretory pathway proteins during the UPR.* Serratia marcescens* and patupilone (EPO 906) are physically assessed materials through clinical trials related to GBM progression. Consequently, our review puts emphasis on the potential of natural products in GBM treatment by regulating multiple malignant cancer-related pathway solving pending problem such as reducing toxicity and side effect.

## 1. Introduction

Glioblastoma (GBM) is the most common and malignant CNS (central nervous system) tumor originating from glial cells [1]. It is one of the most lethal types of brain tumor [2]. During the past 30 years, the therapies for this dreadful disease were researched [3]. The most standard care for GBM is surgical resection followed by radiotherapy and temozolomide- (TMZ-) based chemotherapy [4]. Although the standard treatments for glioblastoma have been introduced, the mean survival period of GBM still remains short, ranging from only 12 to 15 months, and the 5-year survival rate is only 4-5%, indicating that contemporary treatments are not as effective in treating glioblastoma [2, 5]. 

There are mainly two reasons behind the limitations on treating glioblastoma. Firstly, various factors in the pathology of glioblastoma deter current chemotherapies from being fully effective. For example, the highly vascularized network of GBM leads to resistance from conventional chemotherapy. Also, the blood-brain barrier (BBB) makes it difficult to deliver the drug to the cancer, resulting in recurrence without full recovery [6].

Secondly, the drugs used in current chemotherapy of GBM have certain limitations. The limitations include side effects and poor effectiveness. TMZ is observed to have severe side effects, such as myelosuppression [7] and cerebral edema [8]. Also, TMZ showed poor improvement in survival periods (increased survival only for about 2 months) in patients who received treatment with TMZ combined with radiation and tumor resection. Furthermore, since patients die from recurrent tumors, chemoresistance is also a problem [9].

Bevacizumab, which was especially approved in United States, also exhibits adverse events, such as interference in normal blood flow and occurrence of coronary artery disease and peripheral artery disease. Other severe side effects include gastrointestinal perforation, bleeding, allergic reactions, blood clots, and an increased risk of infection [10]. Moreover, some say that the addition of bevacizumab to standard chemotherapy in patients with advanced ovarian cancer is not cost effective [11].

The limitations of current chemotherapy necessitate the need for novel drugs that can be more effectual, induce less side effects, and bring a favorable prognosis. Already, natural products express various potentials, such as enhanced bioavailability and increased stability when forming interaction between active constituents [12]. Also, especially in cancer treatment, traditional oriental herbal medicine is used by patients to improve immunity, since natural killer (NK) cells are activated when drugs are admitted. This leads to inhibition of tumor development and progression, helping the survival of cancer patients [13].

Consequently, we collected recent papers including efficacy for GBM treatment on the grounds of angiogenesis, metastasis, apoptosis, ER stress, ROS, MDR, and miRNA through increased stability, protection from toxicity, enhanced pharmacological activity, improved tissue macrophage distribution, and protection from physical and chemical degradation.

The purpose of this review exhibits scientific accuracy and quality compared to traditional data that is to summarize and organize by standardization, efficacy mechanism, and justification of pharmacokinetic and pharmacological parameter [14–18].

## 2. Apoptotic Effect of Natural Products

Apoptosis is a well-organized programmed cell death, which is induced by various natural products (Table 1) [19]. The methanol extract of* Angelica sinensis* (AS-M) is commonly used in natural product to treat several diseases. AS-M activates both p53-dependent and caspase-independent pathways for apoptosis by inducing cell cycle arrest [20]. Hyperforin (HP), polyphenolic procyanidin B2 (PB-2), and hypericin (HY) are extracts of* Hypericum perforatum* L. (*H*.* perforatum*). HP causes cell death by apoptosis involving a caspase-dependent pathway. PB-2 triggers cytostatic and apoptotic activities in LN229 [21].* Angelica sinensis* has been observed to have biological activities in traditional Chinese medicines. Cell cycle arrest and apoptosis of AS-C induce anticancer effects in GBM. n-Butylidenephthalide (BP) is isolated from the chloroform extract of* Angelica sinensis*. It is a naturally occurring compound, triggering cell cycle arrest and apoptosis in malignant brain cancer. BP has antitumoral activity in GBM cells via mitochondria-dependent apoptosis and PKC signaling which associates upregulation Nur77 [22]. Flavonoid-rich fraction 6 (Fr6) and proanthocyanidins (PAC) fraction are isolated from cranberry presscake and whole cranberry. They have potent anticancer effects, such as inducing cell cycle arrest and apoptosis [23]. TCE, which is a methanol extract of* Tinospora cordifolia*, significantly showed increase of GFAP expression and differentiation in C6 glioma cells [24]. Thymoquinone (TQ), a compound isolated from Nigella sativa seed oil, has autophagic activity via mediating lysosomal membrane permeabilization, as well as caspase-independent apoptotic cell death [25]. Niclosamide is one of the 160 synthetic and natural toxic substances. It inhibits NOTCH-, mTOR-, and NF-kB signaling cascades of pGBM cells [26]. Brazilin, one of the compounds in* Caesalpinia sappan*, enhanced apoptosis in glioma cells with an increase of the ratio of cleaved PARP and a decrease of the expression of caspase-3 and caspase-7 [27]. Δ(9)-Tetrahydrocannabinol (THC) and cannabidiol (CBD) are two main cannabinoids contained in marijuana. THC- and CBD-loaded microparticles showed enhanced apoptosis and reduction of cell proliferation and angiogenesis in mice bearing glioma xenografts [28]. Two resveratrol oligomers, hopeaphenol and r2-viniferin, showed antiproliferative effect in D-GBM cells by inducing caspase-9 and caspase-3/7 activation [29]. wogonin is one of the main compounds of* Scutellaria baicalensis*, which trigger growth arrest as well as apoptosis by generating reactive oxygen species in human glioma cells. It is also shown that wogonin affects DNA damage, p53 regulation, and the suppression of protein synthesis [30]. Both curcumin and chokeberry extract reduce MMP gene expression in order to inhibit invasion and induce apoptosis [31]. Zeng Sheng Ping (ZSP, also known as ACAPHA and antitumor B) is a composition of 6 traditional Chinese herb used in numerous cancers. It also has an effect on medulloblastoma and glioblastoma, inhibiting Notch signaling and reducing expression of stem cell markers [32]. Andrographolide, a compound isolated from Andrographis paniculata, inhibits PI3K/AKT signaling pathway and arrests the G2/M phase, to mediate cell proliferation [33]. 3-Deoxyschweinfurthin B (3dSB) and 3-deoxyschweinfurthin B-like* p*-nitro-bis-stilbene (3dSB-PNBS) are two similar compound which mimics schweinfurthin activity. They induce PARP cleavage and eIF2 phosphorylation and show increase of GRP78 and PDI expression [34]. Jaceosidin, which is isolated from the Chinese herb* Artemisia argyi*, leads glioblastoma cells to apoptosis in the G2/M phase via mitochondrial-caspase-3-dependent pathway [35]. Resveratrol, a natural compound well-known for autophagic activity, develops U87 glioma cells into autophagosome and arrests the cell cycle in S-G2/M phase, although not being related to its cytotoxicity [36]. Tagitinin C, which is isolated from* Tithonia diversifolia* methanolic extract, increases PARP, p-p38, ULK1, and LC3-II expression to autophagy interplay with apoptosis in glioblastoma [37]. 2 (Z)-N-(2-(Dimethylamino)ethyl)-2-(3-((3-oxoisobenzofuran-1(3H)-ylidene)methyl)phenoxy) acetamide (PCH4) is a derivative of n-butylidenephthalide (BP). It mediates the JNK pathway and decreases Nur77 expression [38]. *γ*-Mangostin, a compound of* Garcinia mangostana*, showed apoptotic activity by ROS production, leading to cell damage and ROS-dependent mitochondrial dysfunction [39]. Inositol hexaphosphate (IP6) is a phytochemical, found in corns, cereals, nuts, and high-fiber content foods. IP6 has apoptotic effects that upregulates calpain and caspase-3 activities and downregulates the survival factors BIRC-2 and telomerase in T98G cells [40]. Methyl gallate isolated from* Spondias pinnata* activates ERK1/2 which results in apoptosis [41]. Water extract of* Ruta graveolens* L., commonly known as rue, induces death in different glioblastoma cell lines. Its effects were mediated by ERK1/2 and AKT activation and the inhibition of the pathways, PD98058 and wortmannin, reverting its antiproliferative activity [42]. Oridonin, a natural diterpenoid compound isolated from the traditional Chinese medicine,* Rabdosia rubescens*, induced U87MG glioma cell apoptosis and RNA accumulation in nucleus at 12 h-time point. Before U87MG cell apoptosis, the RanGAP1 protein amount decreased and RanGTP accumulated in nucleus [43]. Deoxypodophyllotoxin (DPT) is a semisynthetic compound derived from the extract of* Dysosma versipellis* (Hance) M.Cheng. G2/M phase arrest by DPT results in cell death. However, DPT failed to downregulate these cell cycle regulatory molecules in SF126 glioblastoma cells and stopped the cell cycle at M phase [44]. Ardipusilloside I (ADS-I) is a natural compound that can be isolated from* Ardisia pusilla* A.DC. It was incorporated into polymer microspheres. ADS-I wafers' biodegradable implants against glioblastoma are associated with a decrease in vascular endothelial growth factor, C-reactive protein, tumor necrosis factor-*α* and interleukin-6, and an increase in interleukin-2 expression [45]. Supercritical CO2 extract of mango ginger (*Curcuma amada* Roxb.) demonstrates anticancer activity in the U-87MG human glioblastoma cell line directly or in synergistic combination with conventional chemotherapeutic drugs. This is related to downregulating the mRNA expression of genes such as STAT3, Bcl-2, and p53 and increases the Bax/Bcl-2 ratio [46]. Curcumin, combined with temozolomide, showed synergy in inhibiting growth of glioblastoma cell line [47].* Hedyotis diffusa* Willd extract inhibits the growth of human glioblastoma cells by inducing mitochondrial apoptosis via AKT/ERK pathways [48]. Icariin and temozolomide demonstrate synergistic anticancer effects in glioblastoma. Icariin inhibited proliferation, induced apoptosis, prevented migration and invasion in U87MG cells, demonstrating the antitumor activities of icariin against GBM [49]. Hispidulin is a naturally occurring flavonoid, which can be extracted from* Saussurea involucrata* Kar. It enhances the antitumor effects of temozolomide in glioblastoma by activating AMPK [50].* Olea europaea* leaf extract improves the treatment response of GBM stem cells by modulating miRNA expression. OLE exhibited apoptosis and necrosis in the GBM cell lines and significantly induced the expression of miR-153, miR-145, and miR-137 and decreased the expression of the target genes of these miRNAs in GSCs [51].* Ficus carica* Latex extract prevents invasion through induction of let-7d expression in GBM cell lines. FCL causes cell death in GBM cells with different responses to TMZ and this effect is synergistically increased in combination with TMZ [52]. Honokiol, a natural bioactive molecular compound isolated from the* Magnolia officinalis*, downregulates STAT3 and activates MAPK, which are involved in the induction of apoptosis in glioblastoma cell line U87. HNK increased expression of Bax and decreased expression of Bcl-2, resulting in downregulation of Bcl-2/Bax ratio and confirming that the intrinsic apoptotic pathway is also involved in HNK-induced apoptosis in U87 cells [53].

Crude extracts from* Rhazya stricta* and* Zingiber officinale* affect growth and proliferation of GBMs. Apoptosis induction was mediated by release of mitochondrial cytochrome c, increased Bax : Bcl-2 ratio, enhanced activities of caspase-3 and caspase-9, and PARP-1 cleavage [54]. Ardipusilloside I, a triterpenoid saponin isolated from* Ardisia pusilla* A.DC, significantly inhibited proliferation of both U373 and T98G glioma cells. The cytotoxic activity of ADS-I is associated with the induction of G2/M arrest and cell apoptosis [55]. Berbamine derivative (BBMD3) inhibits cell viability and induces apoptosis in cancer stem-like cells of human glioblastoma, via upregulation of miRNA-4284 and JNK/AP-1 signaling. BBMD3 also increased phosphorylation of the cJun N-terminal kinase (JNK)/stress-activated protein kinase (SAPK), resulting in increased expression of phosphorylated cJun and total c-Fos [56]. Withaferin A, an oxidative cytotoxic agent, resensitizes temozolomide-resistant glioblastomas via MGMT depletion and induces apoptosis through AKT/mTOR pathway inhibitory modulation [57]. Lycorine, C1, C2-ether derivatives of* Sternbergia lutea*, downregulates activity of highly lipophilic analogues against cancer cells. The derivatization of C1- or C2-hydroxyls as methyl ethers causes a complete loss of activity [58]. Perillyl alcohol (monoterpene alcohol) and limonene, respectively, play an important role in cancer therapy. Both can inhibit tumor progression through downregulation of basal production of VEGF in cancer cells. They also suppress the mevalonate pathway and isoprenylation of small G proteins, leading to tumor regression [59]. The methanol extract of* Angelica sinensis* induces cell apoptosis and suppresses tumor growth in human malignant brain tumors. The AS-M mechanism was found to involve the cyclin/CDK/CKI cell cycle regulatory system and the upregulation of p16 and p53 expression [20]. Thiazolo (5,4-d) pyrimidines displayed significant antiproliferative activity, particularly in leukemia and lung adenocarcinoma cells [60]. Chinese traditional herb Nan-Chai-Hu, the root of* Bupleurum scorzonerifoliu*, has isochaihulactone. Isochaihulactone-induced DDIT3 caused apoptosis by stimulating pERK-independent apoptosis. Used with isochaihulactone in GBM cell lines, it can cause ER homeostasis disruption by increasing inducing DNA damage inducible transcript 3 (DDIT3) and NAG-1 expression. PARP and caspase-3/9/7 are also increased, and Bcl-2 is decreased. The cell cycle arrested at G2/M phase and showed increased apoptosis. DDIT3 expression was independent of 78 kDa glucose-regulated protein (GRP78) and protein kinase RNA-like endoplasmic reticulum kinase (pERK) expression. In in vivo studies, tumor growth was suppressed. Also, in the xenograft model, DDIT3 and caspase-3 overexpression, not pERK expression, were observed in the xenograft model [61]. Cortex lycii radicis is the dried root bark of* Lycium chinense*. Growth inhibition effect on GBM cells was observed from crude extract of Cortex lycii radicis. Kukoamine A (KuA) is a spermine alkaloid derived from it. KuA treatment suppressed proliferation, colony formation, growth of tumors, migration, and invasion of GBM cells. KuA increases apoptotic proteins, Bax, and caspase-3 and decreases antiapoptotic protein Bcl-2. In addition, E-cadherin was increased, and 5-lipoxygenase (5-LOX), CCAAT/enhancer binding protein *β* (C/EBP*β*), N-cadherin, vimentin, twist, and snail+slug were decreased. Cell cycle was arrested in G0/G1 phase, and S phase was reduced in a dose-dependent manner in both U251 and WJ1 cells. On human normal liver cells (LO2), KuA showed less cytotoxicity [62].* Nardostachys jatamansi* Rhizome extract (NJRE) reduced caspase-3, caspase-9, and PARP. NJRE at lower dose (20~40 *µ*g/mL) caused excessive nucleation, mitotic catastrophe, DNA fragmentation, and early apoptosis, while higher dose (60~80 *µ*g/mL) induced late apoptosis and G0/G1 arrest [63]. Myricetin (MYR) is one of the natural herbal flavonoids, which has noticeable anticancer properties with nearly zero side effects. MYR-induced cytotoxicity caused glioblastoma cell death by mitochondrial apoptotic pathway. Treated with MYR, cytochrome c, Bcl-2, MDM2, K-Ras, Raf-1, and ERKs (ERK and pERK) are decreased, and Bax, cleaved caspase-3, caspase-9, and Bad are increased. Pluronic-based micelle encapsulation on MYR (MYR micelles (MYR-MCs)) strengthens the effect of MYR itself [64]. Lemon balm (*Melissa officinalis*) aqueous extract has a number of phenolic compounds, protocatechuic, caftaric, caffeic, ferulic, and cichoric acids and flavonoid luteolin-7-glucoside. At 50 *µ*M–200 *µ*M, it showed cytotoxic effect and initiated apoptotic cell death. The biggest amount of active compounds was extracted when using 70% ethanol and has the highest cytotoxic activity on glioblastoma cells. At lower concentrations, intracellular reactive species was decreased. By contrast, at higher concentration, intracellular reactive species was increased. Rosmarinic acid (RA) can be also be found in the dominant and predominant compound. RA showed cytotoxicity on glioblastoma cells. Its LC50 is 290.5 *µ*M for 24 h and 171.3 *µ*M for 48 h. 80–130 *µ*M of RA caused an antioxidant effect and suppression of the cell proliferation. At higher than 200 *µ*M, RA have a prooxidant effect and initiate necrotic cell death [65]. *β*-Escin is natural compound that is a selective inhibitor of glioblastoma-initiating cells (GIC) viability. *β*-Escin exhibited significant cytotoxicity in nine patient-derived GIC, while no substantial effect on the other human cancer or control cell lines is tested. Furthermore *β*-escin had stronger effect than current clinically used cytotoxic agents at reducing GIC growth. It triggers caspase-dependent cell death and causes a loss of stemness properties. But blocking apoptosis could not reduce the *β*-escin-induced effect in sphere formation or stemness marker activity. This result suggests that *β*-escin directly changes the stem identity of GIC, independent of inducing the cell death [66]. Acori Graminei Rhizoma is used for traditional medicine, which has beneficial effects on CNS disorders. Volatile oil of Acori Graminei Rhizoma (VOA) was tried on human glioblastoma multiforme (GBM) cells. VOA suppressed tumor cell growth greatly and showed very low effect on fibroblasts and human glial HEB cells. By VOA, caspase-dependent apoptosis, and p53/AMPK/mTOR signaling pathway autophagy was observed in p53 wild-type A172 cells, and also caspase-independent apoptosis and mTOR-independent pathway autophagy in p53 mutant U251 cells were examined [67]. Ginsenoside Rg3 significantly inhibits proliferation, arrests the cell cycle, and induces apoptosis in HUVEC through reducing VEGF and Bcl-2 expression by combining temozolomide (TMZ) [68]. Zataria multiflora Boiss (Lamiaceae) (ZM) has antioxidant and anti-inflammation activities. Several compounds like thymol, carvacrol, zatrinal, oleanolic acid, betulic acid, rosmarinic acid, monoterpenoids, sesquiterpenoids, p-cymene, and y-terpinen are found in it; above all, thymol and carvacrol are main compounds. After ZM extract treatment, antiproliferation effect of Ionizing radiation (IR) was strengthened only on human glioblastoma (A172) and it showed insignificant change on human nonmalignant fibroblast cell (HFFF2) [69]. Shikonin is an anthraquinone found from the root of lithospermum. After shikonin treatment on human glioblastoma cells, MMP-2, MMP-9, p-AKT, and p-PI3K decreased. However p-*β*-catenin Y333 against *β*-catenin was reduced significantly in the U87 cells, while it was increased in the U251 cells [70]. Propolis is a natural resinous product collected from various plant sources by honeybees. Prenylflavanone (propolin G) is isolated from Taiwanese propolis (TP). This compound induces apoptosis in brain cancer. Propolin G and TP extract protect cortical neurons against oxidative stress in rat [71]. Curcumin (diferuloylmethane) is a natural compound that can be found in turmeric (*Curcuma longa*). It is a well-known agent that has anticarcinogenic activity in tumor cells. Curcumin induces cell cycle arrest (G1 phase) and it has ERK and JNK MAPK/Elk-1/Egr-1 signal that is required for p53-independent transcriptional activation of p21Waf1/Cip1 in U-87MG glioblastoma cells [47]. Berberine, an isoquinoline plant alkaloid, has been used for the treatment of many diseases. It is isolated from traditional Chinese herbal medicine,* Coptis chinensis*, and* Hydrastis canadensis* [72]. Berberine induces G1 arrest and apoptosis in T98G cells. It is mediated through the disruption of the mitochondrial membrane potential and activation of caspase pathways [73]. Three benzopyrans, 6-isobutyryl-5,7-dimethoxy-2,2-dimethyl-benzopyran, 7-hydroxy-6-isobutyryl-5-methoxy-2,2-dimethyl benzopyran, and 5-hydroxy-6-isobutyryl-7-methoxy-2,2-dimethyl-benzopyran, are isolated from the chloroform extract of* Hypericum polyanthemum*. They induce cell cycle arrest G2/M phase by increasing sub-G1% in U-373MG [74].

## 3. ROS Generation of Natural Products

ROS generation is closely related to apoptosis [75]. Some natural products activated ROS generation in GBM (Table 2). Balanitin-6 (28%) and balanitin-7 (72%) are isolated* Balanites aegyptiaca* which is an African plant of medicinal interest. This compound has anticancer activities via depletion of [ATP]i. It leads to disorganization of actin cytoskeleton [76]. Obtusaquinone (OBT) activate cellular stress pathways and DNA damage via rapid increase in intracellular ROS levels [77]. *γ*-Mangostin in* Garcinia mangostana* induces ROS and activates NK cells [39]. Propolis significantly suppressed cell death and reactive oxygen species production from homocysteine (Hcy), in dose-dependent manner. In an in vivo study, propolis ingestion improved cognitive function from cognitive dysfunction of Hcy which caused hyperhomocysteinemia [78].

## 4. Antiangiogenesis Effect of Natural Products

**Figure 1:**
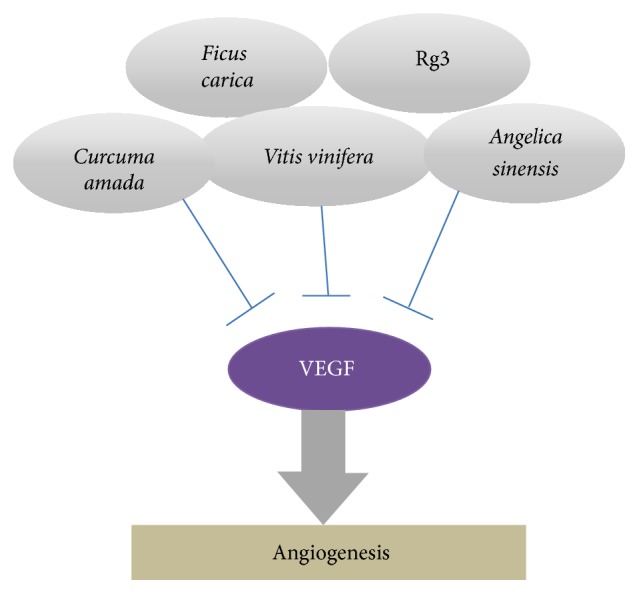
Antiangiogenesis effect of natural products.

A range of natural products exerted antiangiogenesis effect (Table 3 and Figure 1). The antiangiogenesis drug ginsenoside Rg3 (RG3) shows additive effects by combining with low-dose metronomic (LDM) temozolomide (TMZ). Combined use of TMZ with RG3 inhibited proliferation of HUVEC and decreased VEGFA and BCL-2 expression in HUVEC. Also the antiangiogenesis effect was also evaluated in the rat model of orthotopic glioma allograft, based upon markers including relative cerebral blood volume (rCBV) by magnetic resonance imaging (MRI) and microvessel density (MVD)/CD34 staining [68]. Mango ginger (*Curcuma amada* Roxb.) is one of the* Curcuma* species, the popular herbal medicine for anticancer. But it is a less-investigated herb for anticancer properties than other related* Curcuma* species. Supercritical CO2 extract of mango ginger treatment showed antiangiogenesis effect by downregulating VEGF [79]. Red grape skin polyphenolic extract has been issued from its antiangiogenic, anti-inflammatory, and anticancer activity. The extract showed decrease of the tube network formation in HUVEC by Matrigel model. It inhibited S1P- and the VEGF-induced endothelial cell migration [80]. Cannabinoids, the active components of marijuana and their derivatives, are currently investigated due to their potential therapeutic application for the management of many different diseases, including cancer. Specifically, Δ9-tetrahydrocannabinol (THC) and cannabidiol (CBD)—the two major ingredients of marijuana—have been shown to inhibit tumor growth in a number of animal models of cancer, including glioma. Treatment of U87-derived xenografts with THC- or CBD-loaded microparticles or with a mixture of THC and CBD microparticles decreased tumor vascularization as determined by immunostaining with the endothelial cell marker CD31 [28].

## 5. Antimetastasis Effect of Natural Products

**Figure 2:**
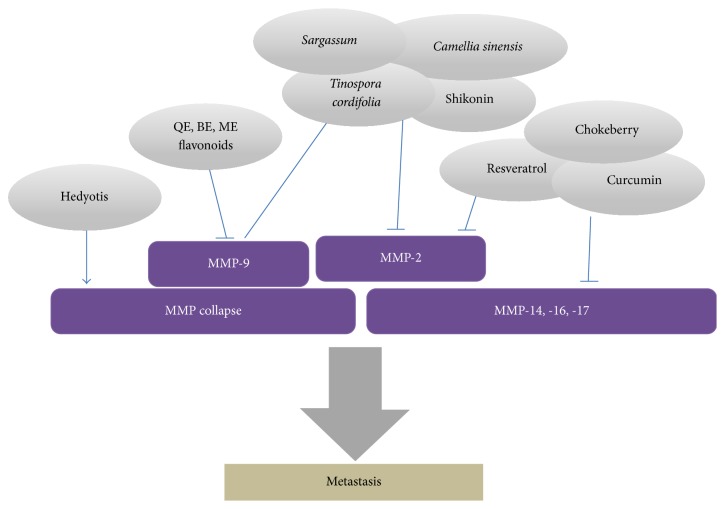
Antimetastasis effect of natural products.

Metastasis is responsible for a majority of cancer-related deaths. Tumor invasion of the surrounding tissue and subsequent metastasis results from a multistep process that includes proteolytic degradation of the surrounding extracellular matrix (ECM), allowing malignant cells to move into and through the ECM and basement membrane. The epithelial-to-mesenchymal transition (EMT) is the crucial step for cancer cells to initiate the metastasis and could be induced by many growth factors. Glioblastoma multiforme (GBM) is one of the most lethal types of tumors and is highly metastatic and invasive. Type IV collagenase matrix metalloproteinases (MMPs), in particular, MMP-2 and MMP-9 and gelatinase A and gelatinase B, respectively, have been found to promote invasion and metastasis of malignant tumors. Various natural products showed antimetastatic effects (Table 4 and Figure 2). Epigallocatechin gallate (EGCG) is the main polyphenol in green tea extract (GTE) [81]. At human glioblastoma (T-98G) cells, MMP-2 and MMP-9 expression decreased with increased concentration of treatment, with the nutrient mixture being most effective, followed by green tea extract and then EGCG [82]. Quercetin (QE), baicalein (BE), and myricetin (ME) are widely used from flavonoids extracted from plants, herbs, and fruits. They induce inhibition of DPPH radical production, PGE2, TPA-induced COX-2 protein, MMP-9 enzyme activity, and peroxide production. QE, BE, and ME can block migration/invasion by GBM cells [83].* Sargassum* (Sargassaceae, Fucales) is a genus of brown seaweed that is found in the ocean. Previous research on* Sargassum* spp. extracts has been reported to exhibit anticancer, antibacterial, antifungal, antiviral, anti-inflammatory, anticoagulant, antioxidant, hepatoprotective, and neuroprotective activities. However, the pharmacological effect of extracts from* Sargassum serratifolium* (*S*.* serratifolium*) has not been thoroughly studied in glioblastoma. Western blot analysis, Τranswell invasion, and wound-healing assays were performed to demonstrate the effects of HES on cell migration and invasion of the U87MG cells. In Western blot analysis, the expression levels of MMP-2 and MMP-9 were decreased in the glioblastoma cells following treatment with HES in a dose-dependent manner [84]. Osthole, a coumarin derivative isolated from the fruit of* Cnidium monnieri* (L.) Cusson, has been widely used for the treatment of skin diseases and gynecopathy. Osthole suggested an anticaner strategy that targets IGF-1 induced EMT. Osthole reversed IGF-1-induced morphological changes, upregulated the expression of epithelial markers, and downregulated the expression of mesenchymal markers. Osthole significantly suppressed the IGF-1-induced upregulation of MMP-2 and MMP-9 in a dose- and time-dependent manner. Moreover, wound-healing assay also showed that osthole could inhibit IGF-1-induced migration of GBM8401 cells [85]. Resveratrol (RES) is a polyphenolic antioxidant found in peanuts, grapes, and red wine, and although parent RES bioavailability might be insufficient to elicit systemic levels commensurate with cancer chemopreventive efficacy, the antioncogenic properties of RES in cells in vitro and in rodent models have been amply documented [86]. RES suppressed the adhesion, invasion, and migration of glioblastoma-initiating cells (GICs) in vitro and in vivo. It inhibited the invasion of GICs via the inhibition of PI3K/Akt/NF-*κ*B signal transduction and the subsequent suppression of MMP-2 expression [86].

## 6. MiRNA Regulation of Natural Products

MiRNA expression is one of important mechanisms in development of cancer. Recently studies on natural compounds reported cancer suppressive effect of altering miRNA expression, which is a new strategy for cancer treatment (Table 5) [51].

*Ficus carica* Latex (FCL) induced the expression of let-7d, targeting epithelial mesenchymal transition of HMGA2 gene, in GBM cells [52].

Shikonin, a natural compound from Chinese medical herb, showed enhanced apoptotic efficacy by overexpressing miR-143 in GSC cells. The antitumor effects of miR-143 were related to BAG3 expression in GSC cells [87]. Berbamine is a natural alkaloid derived from the traditional Chinese medicine, which showed inhibition of cell viability and apoptotic efficacy in GBM stem-like cells by increasing miR-4284 expression [56]. MiRNA modulating effect of* Olea europaea* (OLE) regulates the expression of miRNA including miR-181b, miR-153, miR-145, miR137, and let-7d. By upregulating these miRNAs, OLE induced antiproliferative effects on GBM cells. Furthermore, synergetic effect was shown in combination treatment of OLE and TMZ [88]. Curcumin enhanced cytotoxicity in GBM cells by upregulating miR-146. The regulation of miR-146/NF*κ*B axis sensitized the TMZ-induced cell death in GBM cells [89].

## 7. Multidrug Resistance and Natural Products

Multidrug resistance (MDR) is a major cause of failure in cancer chemotherapies which is presented by numerous cancer cells by withstanding increasing dose of drugs. Consequently, novel compounds derived from natural products are suggested as potential solutions of MDR (Table 6). Withaferin A is a steroidal lactone derived from natural products, demonstrating oxidative mechanism related to AKT/mTOR pathway modulation, MAPK survival, and proliferation pathway in TMZ-resistant GBM cells [57].* Aframomum arundinaceum* extract [90], 8-hydroxycudraxanthone G, cudraxanthone I [91], and sobavachalcone [90] were observed to have hypersensitivity, which means lower drug resistance, to GBM cells.

## 8. Clinical Trials of Natural Production against GBM

Patients with GBM have short survival and most of them develop recurrent or progressive disease after their initial treatments [92]. Two clinical trials on glioblastoma patients were examined (Table 7), but since the clinical trials were progressed no more than phase II, further evaluation of the clinical aspects of these drugs should be reconsidered.

ImuVert is a biologic response modifier derived from* S*.* marcescens* bacteria. A study suggested that ImuVert treatment has minimal toxicity and is well tolerated and contributing to prolonged survival properties in patients newly diagnosed as GBM. The patient survival was slightly prolonged after the treatment with median survival 69 weeks and median time to progression 11 weeks [93]. Another clinical trial with patupilone, which is a natural microtubule-stabilizing cytotoxic agent, showed prolonged survival in GBM patients after their second surgeries. 2 out of 9 patients were recurrence-free after the treatment for 9.75 and 22 months each. Moreover, median survival of all patients was 85 months after their first surgeries. The result suggests that patupilone treatment can be given to recurrent GBM patients before and after surgery safely [92].

## 9. Conclusion

In this review, we categorized precedent studies that encompass various mechanisms of natural products, such as suppression toward apoptosis, angiogenesis, metastasis, ER stress, and MDR, taking into view the standardization of natural product-derived drugs and evaluation of drug doses that display maximum effectiveness [94].

It is well known that GBM is far more difficult to treat than other malignant cancers, mainly due to its pathological properties [95]. Consequently our review puts emphasis on shedding light on the potential of natural products for GBM treatment by solving the impending problem regarding the limitations of current glioblastoma therapy. Certain natural products shown in our review have potent antiglioblastoma properties that have been tested in* in vitro* and* in vivo* laboratory situations. Clinical trials also exhibit compelling effects, although they are still undergoing further evaluation, and their cases are minor in number. More preclinical and clinical studies should be conducted to elucidate the effects and mechanisms of natural products.

## Figures and Tables

**Table 1:** Apoptotic effect of natural products.

Family names	Medical plants	Compounds/extracts	Dose/duration	Target molecules and additional efficacy	Cell lines	References
Hypericaceae	*Hypericum perforatum L. (H. perforatum)*	Hyperforin (HP), polyphenolic procyanidin B2 (PB-2), hypericin (HY)	HP 20 *µ*M for 24 h PB-2 80 *µ*M for 24 h (Annexin V-binding analysis)	Annexin V positive cells	LN229	[21]

Apiaceae	*Angelica sinensis*	n-Butylidenephthalide (BP)	75 *µ*g/mL for 6, 12, 24 h (cell cycle analysis) 75 *µ*g/mL for 24, 48, 72 h (Cell Death Detection Kit, POD) 75 *µ*g/mL for 0, 1.5, 3, 6, 12, 24, 48 h (Western blot analysis)	DBTRG-05MG, DBTRG 8401 (human) ↑p53, p16, Bax, AIF protein induce Fas expression, caspase-8 (dose-dependent), procaspase-9, procaspase-3 ↓cdk2, cdk4, cdk6, cyclin D1, cyclin E, RG2 (rat) ↑p27, Bax, AIF induce Fas expression, caspase-8 (maximum expression at 24 h), procaspase-9, procaspase-3 ↓cdk2, cdk4, cdk6, cyclin D1, cyclin E, p21 cell cycle arrest (G0-G1 phase)	DBTRG-05MG, GBM 8401 (human), RG2 (rat)	[22]

Ericaceae	*Vaccinium macrocarpon* (cranberry)	Flavonoid-rich fraction 6 (Fr6), proanthocyanidins (PAC) fraction	Fr6 concentration: 0, 50, 100, 150, 200, 250, 300 mg/L for 24 and 48 h (cell cycle distribution analysis) (Annexin V, PI) PAC concentration: 0, 20, 40, 60, 80, 100, 120, 140, 160 mg/L for 24, 48 h (cell cycle distribution analysis) (Annexin V, PI)	↑G1 phase ↓S phase cell cycle arrest (G1 phase)	U87MG	[23]

Menispermaceae	*Tinospora cordifolia*	Ethanol extract	250 *μ*g/ml, 350 *μ*g/ml for 72 h	↑GFAP, NCAM, ↓MMP-2,9, cyclin D1, Bcl-xl	C6, U87 MG	[24]

Ranunculaceae	*Nigella sativa*	Thymoquinone	20 *μ*M, 40 *μ*M for 24 h	↑p62, cathepsin B	T98G, U87 MG	[25]

		Niclosamide	1.5 *μ*m/L for 48 h	↓WNT/CTNNB1-, NOTCH-, mTOR-, NF-kB	LN229, T98G, U87MG, U138, and U373 MG	[26]

Fabaceae	*Caesalpinia sappan*	Brazilin	10 *μ*g/ml, 15 *μ*g/ml, 20 *μ*g/ml for 24 h	↑PARP ↓caspase-3, caspase-7	U87 MG	[27]

Cannabaceae	Marijuana (cannabis)	Δ(9)-Tetrahydrocannabinol (THC) and cannabidiol (CBD)	75 mg MPs (biodegradable polymeric microparticles) every 5 days	↓KI67, CD31	U87 MG	[28]

		Hopeaphenol, r2-viniferin	20 *μ*g/ml (hopeaphenol), 100 *μ*g/ml (r2-viniferin) for 120 h	↓caspase-9, caspase-3/7	D-GBM	[29]

Lamiaceae	*Scutellaria baicalensis*	Wogonin	0–100 *μ*M for 24 h	↑ AMPK, p53 ↓mTOR, 4E-BP1 G0/G1 phase arrest	U87 MG, U343 MG, U373 MG, T98G, MCF-10A	[30]

Rosaceae	*Aronia melanocarpa*	Chokeberry extract, curcumin	10 *μ*g/ml (curcumin), 50 *μ*g/ml (polyphenolics from *Aronia melanocarpa)*	↓MMP-2, -14, -16, -17	U373 MG	[31]

(1) Fabaceae (2) Polygonaceae (3) Lamiaceae (4) Asteraceae (5) Rutaceae (6) Dioscoreaceae	*(1) Sophora tonkinensis* *(2) Polygonum bistorta* *(3) Prunella vulgaris* *(4) Sonchus brachyotus* *(5) Dictamnus dasycarpus* *(6) Dioscorea bulbifera*	ZSP (Zeng Sheng Ping)	0, 50, 100 mg/kg/day	↓notch 2, Hes1, CD133	U87 MG, HSR-GBM1 JHH-GBM10, JHH-GBM14	[32]

Acanthaceae	*Andrographis paniculata*	Andrographolide	10 *μ*M	↓PI3K/AKT, caspase-3 G2/M phase arrest	U251, U87 MG	[33]

	*Macaranga schweinfurthii*	3-Deoxyschweinfurthin B (3dSB), 3-deoxyschweinfurthin B-like *p*-nitro-bis-stilbene (3dSB-PNBS)	3dSB (500 nM), 3dSB-PNBS (500 nM), DMP-PNBS (1 M), or Y-27632 (10 M) for 48 h	↑PARP, GRP78, PDI ↓caspase-9	SF-295	[34]

Asteraceae	*Artemisia argyi*	Jaceosidin	100 *μ*M/L for 24 h	↑p53, Bax, cytochrome c, caspase-3 G2/M phase arrest	U87 MG	[35]

		Resveratrol	30 *μ*M for 48 h	↑ Atg5, beclin-1, LC3-II, PI3k class III ↓CD133, OCT4, mTor/AKT/p70S6K S-G2/M phase arrest	U-87 MG, U-251, U-138 MG	[36]

Asteraceae	*Tithonia diversifolia*	Tagitinin C	10 *μ*g/mL for 12 h	↑PARP, p-p38, ULK1, LC3-II	U373 MG	[37]

Apiaceae	*Angelicasinensis*	(Z)-N-(2-(Dimethylamino)ethyl)-2-(3-((3-oxoisobenzofuran-1(3H)-ylidene)methyl)phenoxy)acetamide (PCH4)	50 *µ*g/ml for 24 h	↓Nur77, JNK	DBTRG-05MG, GBM 8401	[38]

Clusiaceae	*Garcinia mangostana*	*γ*-Mangostin	80 *μ*M for 8 h	↑NK cell, ROS ↓PGE2, COX-2, NO	U87 MG, GBM 8401	[39]

	High-fiber foods (such as corns, cereals, legumes, nuts, oil seed, soybean)	Inositol hexaphosphate (IP6)	0.5, 1 mM for 24 h	↑Bax, Bax: Bcl-2 ratio, cytosolic level of cytochrome c, Smac/Diablo (in the cytosol), 80 kD calpain, caspase-9, 85 kD PARP fragment ↓cytochrome c, Smac/Diablo, Bcl-2, BIRC-2, hTERT	T98G	[40]

Anacardiaceae	*Spondiaspinnata*	Methyl gallate	1 to 30 *μ*g/ml for 48 h	ERK1/2 activation, apoptosis	U87MG	[41]

Rutaceae	*Rutagraveolens* L.	*R*. *graveolens* a.e.	1 mg/ml for 24, 48, 72 hours	ERK1/2, AKT activation, apoptosis in A1 mes-c-myc cells	U87MG, C6, U138	[42]

Lamiaceae	*Rabdosiarubescens*	Oridonin	5 *μ*M for 12 h	↓RNA transferation, RanGTP	U87MG	[43]

Berberidaceae	*Dysosmaversipellis*	Deoxypodophyllotoxin (DPT)	30 nM for 72 h	↓Cdc2, cyclin B1, Cdc25c	U87MG, SF126	[44]

Primulaceae	*Ardisiapusilla* A.DC	Ardipusilloside I (ADS-I)	(20 *μ*L, 5 mg/mL) for 4 h	↓vascular endothelial growth factor, C-reactive protein, tumor necrosis factor-*α*, interleukin-6, interleukin-2	rat C6 glioma cells (in vivo)	[45]

Curcuma	*Curcumaamada* Roxb.	Supercritical CO2 extract of mango ginger (CA)	0–100 mg/mL for 48 h	↓STAT3, Bcl-2, mutant p53 expression ↑ratio of Bax/Bcl-2	U87MG	[46]

		Curcumin, temozolomide	7.5 *μ*M for 72 h	↓phosphorylation of cyclin B1, cyclin D1 G2/M arrest	C6, U251MG U87MG	[47]

Rubiaceae	*Hedyotisdiffusa* Willd	HDW extract	0, 4, 8 mg/ml for 24 h	↓Bcl-2/Bax ratio, AKT suppression ↑caspase-3, Bcl-2, Bax and ERK S/G2-M phase arrest, MMP collapse	U87MG	[48]

Berberidaceae	*Epimediiherba*	Icariin	0, 5, 10, 20 *μ*M for 2 h	↓NF-kB, piwil4, Rac1 vasodilator-stimulated phosphoprotein (VASP)	U87MG	[49]

		Hispidulin	10 *μ*M for 2 h	↓Bcl2 ↑AMPK G2 cell cycle arrest	Human GBM SHG44	[50]

Oleaceae	*Oleaeuropaea*	Olea europaea leaf extract (OLE)	1 mg/ml, 2 mg/ml for 24 h	↑ miR-153, miR-145, miR-137	T98G, U-138MG, U-87MG	[51]

Moraceae	*Ficuscarica* Latex	FCL extract	0.125 mg/ml for 24 and 48 h	↓HMGA2, VEGFA ↑HMGA2, VEGFA	T98G, U-138 MG, U-87 MG	[52]

Magnoliaceae	*Magnoliaofficinalis*	Honokiol	10, 20 *µ*g/ml for 12 or 24 h	↓STAT3 signaling, ERK1/2, ↑p38 MAPK signaling pathway G0/G1 phase cell cycle arrest	U87MG, U251, T98G	[53]

Apocynaceae/Zingiberaceae	*Rhazyastricta*, *Zingiberofficinale*	Crude alkaloid (CAERS), flavonoid (CFEZO)	10 *µ*g/mL/for 24, 48, 72 h	↓nuclear NF-kB, p65, survivin, XIAP, cyclin-D1, ↑mitochondrial cytochrome c, Bax : Bcl-2 ratio, activities of caspase-3 and -9, and PARP-1 cleavage, p53, p21, Noxa	U251	[54]

Primulaceae	*Ardisiapusilla* A.DC	Ardipusilloside I	20 *µ*g/mL for 24 h	↑Beclin 1, LC3 arrest at G2/M phase	U373, T98G	[55]

Berberidaceae	*Berberisamurensis*	Berbamine derivative, BBMD3	1 *µ*g/mL/24 h or 48 h	↑miRNA-4284, JNK/AP-1 signaling, caspase-3 and cleavage of poly (ADP-ribose) polymerase (PARP) microRNA-4284 (miR-4284), phosphorylation of the cJun N-terminal kinase (JNK)/stress-activated protein kinase (SAPK)	Cancer stem-like cells (CSCs) from four GBM patients (PBT003, PBT008, PBT022, and PBT030)	[56]

Solanaceae		Withaferin A (WA)	0.025–3 *μ*M for 72 h	↓G2/M cell cycle arrest, phosphorylation of AKT, mTOR, p70 S6K, c-Met, EGFR, Her2	U87MG, U251, T98G	[57]

*Amaryllidaceae*	*Sternbergialutea*	Lycorine		↓actin, CYP3A4 cytostatic effect	B16F10 melanoma (mice)	[58]

	Lavandin, peppermint, spearmint, sage, cherries, cranberries, *Perilla* (*Perillafrutescens*), lemongrass, wild bergamot, gingergrass, savin, caraway, celery seeds//lemon	Perillyl alcohol (monoterpene alcohol) //		Perillyl alcohol: ↑TGF-*β* Limonene: ↓isoprenylation, coenzyme Q synthesis		[59]

Apiaceae	Angelica sinensis	Methanol extract of *Angelicasinensis*	100 *µ*L for 72 h	↑p16 and p53, CDK inhibitors cell cycle arrest at the G0-G1 phase	DBTRG-05MG, BALB/3T3	[20]

	4,6-Dichloro-5-aminopyrimidine	Thiazolo[5,4-d] pyrimidines	48 h		T98G	[60]

Apiaceae	*Bupleurum scorzonerifolium (Nan-Chai-Hu)*	Isochaihulactone	No concentration (all cell ER stress Western) 80 *μ*M for 0, 24, 48 h (cell cycle) 20, 40, 80 *μ*M for 24 h (Annexin V-PI double stain) 50, 200 mg/kg daily for 30 d (in vivo)	↑DDIT3, NAG-1, PARP, caspase-3/9/7 ↓pERK, Bcl-2 cell cycle arrest G2/M phase, subG1 population increase (naturally pERK induce DDIT3. But DDIT3 increased with low pERK. There is new pathway to increase DDIT3)	GBM cell lines 8401, 8901, U87MG, G2T, 131TXM, 1XM, RG2, GL261 (each cell for ER stress Western, 2 cells for cell cycle)	[61]

Solanaceae	*Lycium chinense (Lycii radicis [Cortex])*	Kukoamine A	0, 5, 10, 20 *μ*M for 48 h (cell cycle) 40, 60, 80 *μ*M for 48 h on U251; 10, 20, 30 *μ*M for 48 h on WJ1 (Annexin V-PI double stain, cell activation observed, Western)	↑Bax, caspase-3, E-cadherin ↓5-Lipoxygenase (5-LOX), Bcl-2, CCAAT/enhancer binding protein *β* (C/EBP*β*), N-cadherin, vimentin, twist and snail+slug Proliferation, colony formation, migration, invasion, growth of tumors all decreased, cell cycle arrested G0/G1 phase, less cell cycle S phase, less cytotoxic for C6	Human GBM cells U251 & WJ1, rat glioma cells (C6)	[62]

Valerianaceae	*Nardostachys jatamansi [Rhizome]*	N/A	0, 20, 40, 60, 80 *µ*g/mL for 24, 48, 72 h (cell counting, AO/EB Dual Fluorescence Staining) 0, 20, 40, 60, 80 *µ*g/mL for 24 h (cell cycle analyses) 0, 20, 40, 60, 80 *µ*g/mL for 1 week (clonogenic assay) 10–70 *µ*g/mL for 24 h (DNA Fragmentation) No concentration measured for 24 h (Western immunoblotting)	↓caspase-3/9, PARP cell shrinkage, membrane blebbing, echinoid processes, pyknosis, myorrhexis, low density (~40 *µ*g/mL) early apoptosis, high density (60 *µ*g/mL) late apoptosis, G0/G1 arrest [60, 80 *µ*g/mL]	U87M (every experiment), U373MG	[63]

N/A	*N/A*	Myricetin	200 *μ*M for continuous time (over 90 h) (real-time cell analyser instrument) 25, 50, 100, 200 *μ*M for 24 h (Western)	↑Bax, cleaved caspase-3, caspase-9, Bad ↓cytochrome c, Bcl-2, MDM2, K-Ras, Raf-1, ERK, pERK	DBTRG-05MG, U251, U87MG (DBTRG-05MG for every experiment, 3 cells for real-time cell viability)	[64]

Lamiaceae	*Melissa officinalis*	Rosmarinic acid (RA) // luteolin-7-glucoside, caffeic acid, rosmarinic acid, protocatechuic acid, caftaric acid, ferulic acid, cichoric acid, Dulbecco's modified Eagle's medium (DMEM), Ampliflu Red, 2′,7′-Dichlorofluorescin diacetate (DCFH2-DA), 3-(4,5-dimethylthiazol2-yl)-2,5-diphenyl-tetrazolium bromide (MTT), HPLC grade acetonitrile, trifluoroacetic acid (TFA)	0, 50, 100, 140, 170, 200, 250, 300, 400 *μ*M for 24, 48 h (RA cell viability) 0, 50, 100, 140, 170, 200 *μ*M for 24, 48 h (N1 cell viability) 0, 10, 25, 40, 50, 75, 100 *μ*M for 24, 48 h (N2, N3 cell viability) (highest density is 0% survive)	↑intracellular reactive species (RS) (high density) ↓intracellular reactive species (RS) (low density) cell proliferation decrease, at low density cell viability increase, at middle density apoptosis & antioxidant effect, at high density prooxidant effect & necrosis RA < Aqueous < Ethanolic 40% < Ethanolic 70% (effect) (pure RA, aqueous (N1), ethanolic 40% (N2), ethanolic 70% (N3), 4 kinds compared)	C6 rat glioblastoma	[65]

N/A	*N/A*	*β*-Escin [temozolomide (TMZ)]	0, 2, 4, 6, 8, 10 *μ*M for 48 h (cell viability) 0, 2, 4, 6, 8, 10 *μ*M *β*-escin + 0, 2, 4, 6, 8, 10, 50, 100 *μ*M TMZ for 48 h (*β*-escin, TMZ combined cell viability) No measurement of concentration for 24 h (Western only PARP-1)	↑cleaved PARP-1 synergy with TMZ	(Patient-derived) glioblastoma-initiating cells (GIC), U87MG	[66]

Araceae	*Acori graminei [Rhizoma]*	N/A	0, 50, 100 *μ*g/mL for 48 h on A172; 0, 100, 200 *μ*g/mL for 48 h on U251 (Annexin V-PI double stain) 0, 25, 50, 100 *μ*g/mL for 48 h on A172; 0, 50, 100, 200 *μ*g/mL for 48 h on U251 (Western)	↑Bax, cleaved caspase-3/8/9, mTOR (A172 only), LC3II/I, atg5, beclin-1, p-AMPK (both) ↓Bcl-2, p-p70S6K, p-mTOR (A172 only), p62 (both) Etop inhibition pathway is contrasted with each experiments dependent/independent confirm	A172, U87MG, U251, U118, 4 cell MTT (all experiments)	[67]

Araliaceae	*Panax ginseng*	Ginsenoside Rg3 [temozolomide (TMZ)]	10, 20, 40, 80, 120, 180 *μ*g/mL TMZ or RG3 for 24, 48, 72, 96, 120, 144 h; 10, 80, 180 *μ*g/mL TMZ&RG3 1 : 1 for 24, 48, 72, 96, 120, 144 h (proliferation inhibition)	↓VEGF Synergy with TMZ	Primary human umbilical vein endothelial cells (HUVECs), rat C6 glioma cell	[68]

Lamiaceae	*Zataria multiflora*	Thymol, carvacrol	0, 25, 50, 100, 150, 200 *μ*g/ml for 2 h and 0, 3, 6 Gy Ionizing radiation (IR). After IR incubated for 48 h in fresh medium (MTT)	ZM treatment strengthen IR antiproliferation	A172	[69]

Boraginaceae	*Lithospermum*	Shikonin	2.5, 5, 7.5 *μ*mol/L for 0, 12, 24, 36, 48, 72 h (Cell proliferation) 0, 2.5, 5, and 7.5 *μ*mol/L for 0, 24, 48 h (scratch wound-healing assays) 2.5, 5, 7.5 *μ*mol/L for 48 h (Western)	↑p-*β*-catenin Y333 (U251 only) ↓MMP-2, MMP-9, p-AKT, p-PI3K (both), p-*β*-catenin Y333 (U87 only) scratch wound-healing assay: higher density shikonin less regrowth	U87MG, U251	[70]

Zingiberaceae	*Curcuma longa*	Curcumin (diferuloylmethane) [temozolomide (TMZ)]	20, 50, 100 *μ*M curcumin for 72 h 100, 300, 500 *μ*M TMZ for 72 h (MTT)	↑N/A ↓N/A Synergy with TMZ	U87MG	[89]

	Taiwanese propolis (Erhmei (in central Taiwan), Fangliao (in southern Taiwan))	Prenylflavanone (propolin G), Taiwanese propolis (TP)	0, 2.5, 5, 10 *µ*g/mL/72 h (cell cycle analysis). 7.5 *µ*g/mL/24 h (morphological analysis of apoptotic cells). 12.5 *µ*g/mL/0, 4, 5, 6, 7 h (activity of caspase. Rat C6 glioma). 0, 2.5, 5, 7.5, 10 *µ*g/mL/48 h (Western blotting assay). 2.5, 5, 10, 20, 40 *µ*M with 1.0 mL of 0.3 mM DPPH in methanol (DPPH free radical scavenging activity)	↑sub-G1 cell population, caspase-3, caspase-8, caspase-9, cleavage form of PARP (85 kDa), p21waf1, ↓ procaspase-3, procaspase-8, procaspase-9, Bid, cyclin B1, cyclin D1, EC50 (20.5 *µ*M)/caspase-dependent signal pathway, mitochondrial-dependent pathway, modulation of cell cycle regulators' gene expression, ROS	rat C6 glioma, DBTRG-05MG (human)	[71]

		Curcumin	20 *µ*mol/L/12, 24 h (cell cycle analysis). 0, 5, 10, 15, 20, 25 *µ*mol/L/2 h (Western blot). 20 *µ*mol/L/0, 0.25, 0.5, 1, 2, 4, 6, 12, 24, 48 h (Western blot). 20 *µ*mol/L/0, 15, 30, 60, 120 min (Western blot). 20 *µ*mol/L/0, 5, 15, 30, 60 min (Western blot)	↑G1 phase, p21, Egr-1, phosphorylated ERK1/2, JNK1/2, p38, phosphorylated Elk-1, ↓S phase, G2-M phase, cyclin D1/cell cycle arrest (G1 phase), ERK and JNK MAPK/Elk-1/Egr-1 signal cascade (p53-independent transcriptional activation of p21Waf1/Cip1)	U-87MG	[47]

Ranunculaceae	*Coptischinensis*, *Hydrastiscanadensis*	Berberine	50, 75, 100, 150 *µ*g/ml/48 h (cell cycle analysis). 0, 50, 100, 150, 200 *µ*g/ml/24 h (Western blot, caspase-3 colorimetric protease assay)	↑G1 phase, p27, Bax, caspase-9, caspase-3, PARP, ↓G2/M phase, S phase, CDK2, CDK4, cyclin D, cyclin E, Bcl-2 family, procaspase-9/cell cycle arrest (G1 phase), apoptosis (disruption of the mitochondrial membrane potential, activation of caspase pathways)	T98G	[72]

	*Hypericum polyanthemum*	Three benzopyrans (6-isobutyryl-5,7-dimethoxy-2,2-dimethyl-benzopyran (1), 7-hydroxy-6-isobutyryl-5-methoxy-2,2-dimethyl benzopyran (2), 5-hydroxy-6-isobutyryl-7-methoxy-2,2-dimethyl-benzopyran (3))	10 *µ*g/ml/96 h (cell cycle analysis)	↑% sub-G1/cell cycle arrest (G2/M phase)	U-373MG	[74]

**Table 2:** ROS generation of natural products.

Family names	Medical plants	Compounds/extracts	Dose/duration	Target molecules and additional efficacy	Cell lines	References
Zygophyllaceae	*Balanites aegyptiaca*	Balanitin-6 (28%), balanitin-7 (72%)	830 nM for 24, 48, 72 h	↓ATP (disorganization of actin cytoskeleton)	U373	[76]

		Obtusaquinone	(In vitro) 5 *µ*M for 24 h (in vivo) 7.5 mg/kg for 21 days	↑ROS, p53, caspase 3/7 ERK pathway	Gli36, U87 MG, U251, GBM8, GBM11/5, VU147	[77]

Clusiaceae	*Garcinia mangostana*	*γ*-Mangostin	80 *μ*M for 8 h	↑NK cell, ROS ↓PGE2, COX-2, NO,	U87 MG, GBM 8401	[39]

N/A	Brazilian propolis (Pro) (not specific plant)	[Homocysteine (Hcy)] to provoke cognitive dysfunction	100 *μ*M DL-Hcy + 0, 0.2, 0.4, 0.8, 1.6, 3.2 *μ*g/mL for 72 h (intracellular ROS detection) (after age 12 weeks mice + 1 week adaption) 0% Pro + Hcy(x), 0% Pro + Hcy(o), 0.25% Pro + Hcy(o), 0.05% Pro + Hcy(o) for 5, 14, 27 week (animal experiment) 0, 100 *μ*M Hcy + 0, 0.27, 1.38 *μ*g/mL Pro (final concentration each) for 0, 7, 11, 14, 17, 21, 24 days	↑relative fluorescence intensity ↓relative ROS intensity, in vivo Plasma Hcy concentrations, in vitro amyloids formation Propolis intake made rats recovered from Hcy induced cognitive dysfunction	Neuroblastoma SH-SY5Y, glioblastoma U-251MG	[78]

**Table 3:** Antiangiogenesis effect of natural products.

Family names	Medical plants	Compounds/extracts	Dose/duration	Target molecules and additional efficacy	Cell lines	References
Araliaceae	*Panax ginseng*	Ginsenoside Rg3	0–180 *μ*g/ml for 72 h (in vitro) 10 mg/kg/day for 8 days (in vivo)	↓VEGF, Bcl-2 (HUVEC mRNA), VEGFA, MVD Inhibit HUVEC proliferation, less increase rCBV	Rat C6 glioma cells	[68]

Zingiberaceae	*Curcuma amada *	Supercritical CO2 extract	0–100 *μ*g/ml for 72 h 0–20 *μ*g/ml for 24 h	↓VEGF mRNA, VEGF	U87MG	[79]

Vitaceae	*Vitis vinifera*	Red grape skin polyphenolic extract	0–25 *μ*g/ml for 24 h	↓tube network formation, VEGF, S1P, ERK, p38/MAPK phosphorylation, S1P-induced PAF synthesis	U87MG	[80]

Cannabaceae	*Cannabis sativa*	Δ9-Tetrahydrocannabinol, cannabidiol	7.5 mg/kg/day for 22 days	↓tumor vascularization (CD31 immunostaining)	U87MG	[28]

**Table 4:** Antimetastasis effect of natural products.

Family names	Medical plants	Compounds/extracts	Dose/duration	Target molecules and additional efficacy	Cell lines	References
Theaceae	*Camelliasinensis* (green tea)	Vitamin C (as ascorbic acid and as Mg, Ca, and palmitate ascorbate) 700 mg; L-lysine 1000 mg; L-proline 750 mg; L-arginine 500 mg; N-acetyl cysteine 200 mg; standardized green tea extract (80% polyphenol) 1000 mg; selenium 30 *μ*g; copper 2 mg; manganese 1 mg	0–1000 *μ*g/ml	↑TIMP-2 ↓MMP-2, MMP-9, uPA	LN-18, T-98G, A-172	[81]

Theaceae	*Camellia sinensis*	Epigallocatechin gallate (EGCG)	50–500 *μ*g/ml	↓-2, MMP-9	T-98G	[82]

Sargassaceae	*Sargassum serratifolium*	Hexane, ethanol, ethyl extract	5–15 *μ*g/ml for 12–24 h	↓ MMP-2, MMP-9, C-Raf, MEK, ERK, phospho-ERK, wound area, invasive cells	U87MG	[84]

Apiaceae	*Cnidium monnieri*	Osthole	20–80 *μ*M for 24 h	↓MMP-2, MMP–9 Inhibit IGF-1-induced EMT	GBM8401	[85]

	*Peanuts*, *grapes*, *red wine*	Resveratrol	5–20 *μ*M for 48 h	↓MMP-2, NF-*κ*B pathway, PI3K/AKT signaling pathway	Glioblastoma-initiating cells (GICs)	[86]

	Flavonoids (plants, herbs, fruits)	Quercetin (QE), baicalein (BE), myricetin (ME)	0–50 *μ*M for 30 min	↓ ERK-activated COX-2/PGE2, MMP-9	U87MG	[83]

**Table 5:** miRNA regulation of natural products.

Family names	Medical plants	Compounds/extracts	Dose/duration	Target molecules and additional efficacy	Cell lines	References
Oleaceae	*Olea europaea*	Oleuropein	1 mg/ml, 2 mg/ml for 24 h	↑miR-153, miR-145, miR-137	T98G, U-138MG, U-87MG	[51]

Moraceae	*Ficus carica*	Protocatechuic acid	0.25 mg/ml for 24 h	↑let-7d, VEGF ↓neovascularization	T98G, U-138 MG, U-87 MG	[52]

Boraginaceae	*Lithospermum erythrorhizon*	Shikonin	2 mg/kg for 24 h	↑miR-143, BAG3	GSC	[87]

Berberidaceae	*Berberis amurensis*	Berbamine	5 mM for 24 h	↑caspase-3, PARP, miR-4284, JNK1, JNK 2, SAPK, phosphorylated c- Jun, total c-Fos apoptosis, JNK-c-Jun/AP-1 signaling pathway	PBT003, PBT008, PBT022, PBT030	[56]

Oleaceae	*Olea europaea*	Oleuropein	1 mg/ml for 24 h, 48 h	miR-181b, miR-153, miR-145, miR-137, ↑let-7d	T98G	[88]

Zingiberaceae	*Curcuma longa*	Curcumin (diferuloylmethane)	20 *μ*M for 72 h	↑miR-146a ↓NF-*κ*B	U-87 MG	[89]

**Table 6:** Multidrug resistance and natural products.

Family names	Medical plants	Compounds/extracts	Dose/duration	Target molecules and additional efficacy	Cell lines	References
Solanaceae	*Withania somnifera*	Withaferin A	2.5 *μ*M, 1 *μ*M for 24 h	↑ERK1/2, HSP32, HSP70 AKT, mTOR, p70 S6K, c-Met, EGFR, ↓Her2, HSF1 Oxidative stress, heat shock response, AKT/mTOR pathway, MGMT	U87, U251, T98G	[57]

Zingiberaceae	*Aframomum arundinaceum*	Methanol extract	40 *μ*g/mL for 72 h	Collateral sensitivity (hypersensitivity)	U87MG.ΔEGFR	[90]

Clusiaceae	*Garcinia nobilis*	8-Hydroxycudra-xanthone G, cudraxanthone I	22.49 *μ*M for 24 h	Collateral sensitivity (hypersensitivity)	U87MG	[91]

Moraceae	*Dorstenia barteri*	Isobavachalcone	23.78 *μ*M for 24 h	Collateral sensitivity (hypersensitivity)	U87MG	[90]

**Table 7:** Clinical trial of natural products.

Phase	Title	Current state	Family names	Medical plants	Compounds/extracts	Additional therapy	Evaluation	Survival	Patients	References
Phase 1, Phase 2	Patupilone (EPO 906) in patients with recurrent or progressive glioblastoma multiforme prior to and after secondary resection: an open-label phase I/II trial	Completed	Myxobacterium	*Sorangium cellulosum*	Patupilone	Salvage treatment after patupilone consisted of bevacizumab, CCNU, a second RT round or surgery	Progression-free survival (PFS), overall survival (OS) at 6 months, patupilone concentration in tumor tissue/toxicity, patupilone concentration in plasma and translational analyses for predictive biomarkers, Aschen Aphasia Test, Rey Auditory-Verbal Learning Test, Rey Visual Design Learning Test, Rey-Osterrieth Complex analyses for predictive biomarkers	Median 85 weeks/median PFS 6 weeks	9 patients/age 42–68 yrs	[92]

Phase 2	Clinical trial of *Serratia marcescens *extract and radiation therapy in patients with malignant astrocytoma	Completed	Enterobacteriaceae	*Serratia marcescens*	ImuVert	Radiation therapy	Physical examination, KPS, assessment of weight, CBC count with differential, coagulation profile, serum chemistries, CT, MRI, thallium SPECT, time to progression of tumor	Median survival 69 weeks/median time to progression 11 weeks	11 patients/newly diagnosed glioblastoma multiforme (GBM)/age 42–69 yrs	[93]
